# Inhibition of Enzymes Involved in Neurodegenerative Disorders and A*β*_1–40_ Aggregation by *Citrus limon* Peel Polyphenol Extract

**DOI:** 10.3390/molecules28176332

**Published:** 2023-08-30

**Authors:** Rosaria Arcone, Antonio D’Errico, Rosarita Nasso, Rosario Rullo, Annarita Poli, Paola Di Donato, Mariorosario Masullo

**Affiliations:** 1 Dipartimento di Scienze Motorie e del Benessere, Università degli Studi di Napoli “Parthenope”, Via Medina, 40, 80133 Napoli, Italy; rosaria.arcone@uniparthenope.it (R.A.); antonio.derrico002@studenti.uniparthenope.it (A.D.); rosaritanasso@gmail.com (R.N.); 2 ISPAAM, Consiglio Nazionale delle Ricerche, Piazzale Enrico Fermi, 1, 80055 Portici, Italy; rosario.rullo@cnr.it; 3 ICB, Consiglio Nazionale delle Ricerche, Via Campi Flegrei, 34, 80078 Pozzuoli, Italy; annarita.poli@icb.cnr.it (A.P.); pdidonato@uniparthenope.it (P.D.D.); 4 Dipartimento di Scienze e Tecnologie, Università degli Studi di Napoli “Parthenope”, Centro Direzionale Isola C4, 80143 Napoli, Italy

**Keywords:** *Citrus limon* peel polyphenols, Alzheimer’s disease (AD), Parkinson’s disease (PD), cholinesterase inhibitor, monoamine oxidase (MAO) inhibitor, A*β*_1–40_ aggregation, superoxide dismutase (SOD) inhibitor, neuroprotection

## Abstract

Alzheimer’s (AD) and Parkinson’s diseases (PD) are multifactorial neurogenerative disorders of the Central Nervous System causing severe cognitive and motor deficits in elderly people. Because treatment of AD and PD by synthetic drugs alleviates the symptoms often inducing side effects, many studies have aimed to find neuroprotective properties of diet polyphenols, compounds known to act on different cell signaling pathways. In this article, we analyzed the effect of polyphenols obtained from the agro-food industry waste of *Citrus limon* peel (LPE) on key enzymes of cholinergic and aminergic neurotransmission, such as butyryl cholinesterase (BuChE) and monoamine oxidases (MAO)-A/B, on A*β*_1–40_ aggregation and on superoxide dismutase (SOD) 1/2 that affect oxidative stress. In our in vitro assays, LPE acts as an enzyme inhibitor on BuChE (IC_50_ ~ 73 µM), MAO-A/B (IC_50_ ~ 80 µM), SOD 1/2 (IC_50_ ~ 10–20 µM) and interferes with A*β*_1–40_ peptide aggregation (IC_50_ ~ 170 µM). These results demonstrate that LPE behaves as a multitargeting agent against key factors of AD and PD by inhibiting to various extents BuChE, MAOs, and SODs and reducing A*β*-fibril aggregation. Therefore, LPE is a promising candidate for the prevention and management of AD and PD symptoms in combination with pharmacological therapies.

## 1. Introduction

In recent years, the improvements in life expectancy have led to an increase in aging-related diseases, including neurodegenerative diseases [1]. In the elderly population, Alzheimer’s disease (AD) and Parkinson’s disease (PD) represent the major neurodegenerative diseases of the Central Nervous System (CNS) that induce a progressive neuronal death and neurological dysfunctions causing both a loss of cognitive and motor functions, leading to physical disability [2]. AD and PD are multifactorial disorders that are characterized by an alteration in neurotransmitter metabolism and formation of protein aggregates [3]. The main clinical features of AD are correlated to the abnormal amyloid beta (A*β*) protein plaques around neurons and neurofibrillary tangles (NFT) of hyperphosphorylated tau in the brain [4], leading to irreversible degeneration of cholinergic neurons and a concomitant decrease in acetylcholine (ACh) levels in the hippocampal and cortical regions [5,6].

Two major enzymes, acetylcholinesterase (AChE) and butyrylcholinesterase (BuChE), hydrolyze ACh and, up to now, pharmacological approaches for the treatment of AD mainly aim to restore acetylcholine (ACh) level in the brain through the administration of specific inhibitors (AChEI and BuChI) [7,8,9,10,11,12].

In PD, pathological hallmarks are represented by the presence of intracellular inclusions of aggregated α-synuclein defined Lewy bodies and death of dopaminergic neurons in the substantia nigra, modulating human movement [13].

AD and PD also show an impaired metabolism of amine neurotransmitters, such as serotonin (5-HT), norepinephrine (NE), dopamine (DA) and the neuromodulator phenylethylamine (PEA) that involves monoamine oxidase (MAO)s activity [14,15].

In humans, MAO are detected as two isoenzymes, MAO-A and MAO-B, that are distinguished for different substrate specificity [16], neuronal or astroglial expression and specific brain localization [17]. MAO-A and MAO-B play different roles since MAO-A regulates dopamine levels whereas MAO-B regulates tonic GABA levels [18]. In addition, MAO-B also contributes to the amyloid beta (A*β)* aggregation by altering the cleavage of the amyloid precursor protein (APP) through the modulation of γ-secretase [19]. MAO-B is overexpressed in the brain of AD patients with the impairment of cognitive function, many efforts have been devoted to identifying synthetic or natural MAO inhibitor (MAO-I) to be used against AD and PD. Based on the different substrate specificity of MAOs, specific inhibitors have been identified, also derived from natural products [20]. In particular, clorigiline and moclobemide have been identified as MAO-A inhibitors, whereas selegiline, rasguiline, safinamide and KDS 2010 specifically inhibit MAO-B [18,21].

In the etiology of AD and PD, it is well known that inflammation and oxidative stress [22,23] impair the production of reactive oxygen species (ROS) and the antioxidant defense system [24,25,26], leading to neuroinflammation and neuronal death. Oxidative stress conditions can be counteracted by the endogenous antioxidant defense system that includes enzymes able to inactivate ROS, such as superoxide dismutases (SODs) [27,28,29,30]. SODs are a multigene enzyme family that catalyzes the dismutation reaction of superoxide anion into oxygen and hydrogen peroxide [31]. For this reason, SODs are also ROS producers, since the reaction leads to molecular oxygen and hydrogen peroxide, another toxic compound, although less reactive than superoxide. In humans, three isoforms of SOD are expressed and, based on the metal co-factor used by the enzyme and the cell localization, these are classified into SOD1, a dimer containing copper and zinc (Cu-Zn SOD), located in the cytosol and in the mitochondrial intermembrane space [32]; SOD2, known as manganese superoxide dismutase (Mn SOD), a tetrameric isoform with exclusive mitochondrial localization considered the main defense barrier in oxygen metabolism [33,34]; and SOD3, a tetrameric copper–zinc enzyme that is an extracellular superoxide dismutase (Ec SOD), synthesized only by some cell types, in particular fibroblasts and endothelial cells, from which it is expressed on the cell surface in association with heparan sulfates [35].

Up to now, drugs used in therapy against AD and PD include cholinesterase inhibitors and NMDA receptor antagonist, such as rivastigmine, galantamine, and donepezil, which demonstrated a good efficacy and a low incidence of adverse effects. In particular, rivastigmine, an inhibitor of each cholinesterase was able to ameliorate cognitive functions in AD [9]. However, these drugs can ameliorate cognitive and motor disfunctions, but they cause hepatic and gastrointestinal side effects [36]. In addition, pharmacologic treatments cannot prevent or reverse the progression of neurodegenerative diseases. Therefore, a great effort has been devoted to identifying compounds able to prevent or delay the insurgence of the disease. Furthermore, because AD and PD are multifactorial diseases, pharmacological strategies for their treatment aimed for a multitargeting approach that provides the identification of ligands able to interact and affect the activity of two or more molecular targets [11,37,38].

Not only synthetic drugs, but also natural active compounds from plants, such as flavonoids, anthocyanins, alkaloids, chalcones, coumarins, xanthones, anthraquinones, terpenes, etc., have been identified either to treat various diseases or for the development of new drugs [39,40]. Many compounds can act as both antioxidant or inhibitor of enzymes involved in AD and PD [41,42,43,44]. Among plant compounds, dietary polyphenols, known for their antioxidant properties, also exert various anti-inflammatory and neuroprotective effects [45,46,47]. A polyphenol-rich diet or supplementation with natural antioxidants, such as vitamin and flavonoid supplements, can prevent and/or delay neurological disorders and their symptoms [46,48].

The Mediterranean diet, rich in polyphenols, can prevent or delay these age-related cognitive and movement disfunctions [45,49], and these beneficial effects have been associated to the high polyphenol content [50]. Polyphenol extract from *Annurca* apple flash inhibits in vitro AChE and MAOs enzyme activity [51]. In addition, residues of citrus peels from the waste of agro-food industry represent a suitable source for obtaining biologically active polyphenols [52,53] that show antioxidant and neuroprotective properties [54,55]. This neuroprotective effect has also been demonstrated by in vivo model studies [56,57,58] and it has been attributed to the ability of polyphenols to cross the blood–brain barrier [59]. In our previous studies, we have demonstrated that *Citrus limon* peel extract (LPE) reduces the interleukin-6-dependent invasiveness of human cancer cells through the STAT-3 signaling pathway and it also inhibits the in vitro activity of AChE [60,61], showing chemo-preventive and neuroprotective effect [62,63].

These results prompted us to explore further the neuroprotective properties of LPE. To this aim, we analyzed the effect of LPE on key enzymes involved in neurodegeneration, such as BuChE, MAO-A/B and its ability to inhibit the A*β*_1–40_ in vitro aggregation. In addition, we also determined the effect of LPE on SODs, that play a major role in the antioxidant defense system. The results obtained show that LPE inhibits the activity of these enzymes and counteracts the beta-amyloid aggregation.

## 2. Results

### 2.1. Effect of LPE on BuChE Activity

**Figure 1:**
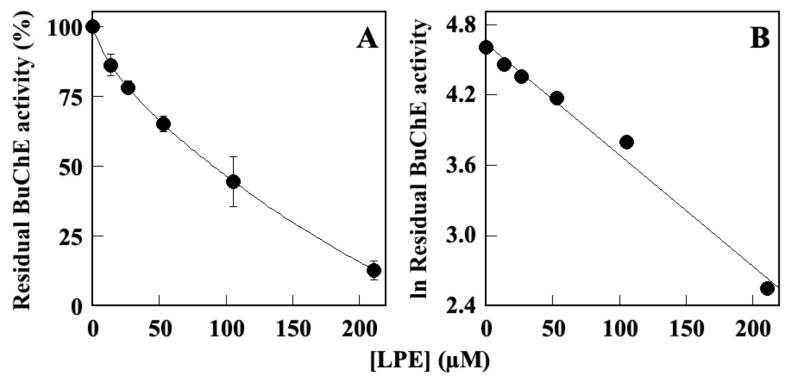
Effect of LPE on in vitro BuChE activity. (**A**) BuChE residual activity was assayed in the absence or in the presence of the indicated concentrations of LPE, as reported in the Methods section. (**B**) Data were analyzed according to a first-order behavior and the IC_50_ value was calculated from the slope of the linear regression (r^2^ = 0.986).

Because cholinesterase inhibitors are used as major drugs in the treatment of Alzheimer’s and Parkinson’s diseases [64,65,66], LPE has been subjected to in vitro enzyme assays to explore its effect on BuChE. In our previous study [60,67], we demonstrated that LPE was able to inhibit AChE activity in a dose-dependent manner with an IC_50_ value of 101 µM. Therefore, in this study, we determined the effect of LPE on in vitro BuChE activity, which allows an accurate determination of the specificity in the inhibition power. The results (Figure 1) indicated that LPE exhibited a concentration-dependent BuChE inhibitory activity (Figure 1A). The analysis of the data by a semilogarithmic plot (Figure 1B) allowed the determination of the inhibitor concentration required to obtain inhibition of half of the activity (IC_50_) corresponding to 72.9 ± 1.5 µM.

### 2.2. Effect of LPE on Aβ_1–40_ Self-Aggregation

One of the main objectives in the prevention of the onset of AD is the prevention of A*β*_1–40_ self-aggregation, leading to neuronal death and alteration in the metabolism of neurotransmitters.

**Figure 2:**
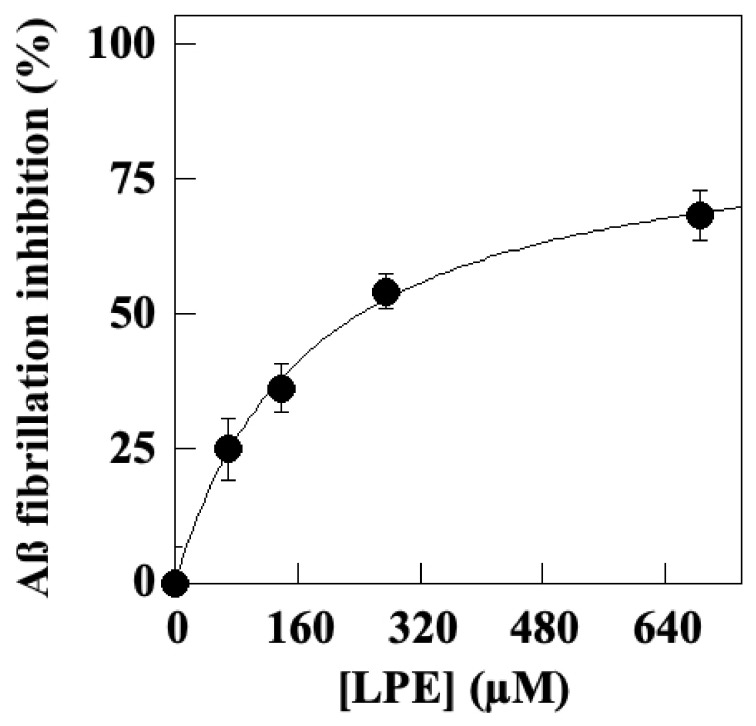
Effect of LPE on in vitro aggregation of A*β*_1–40_ amyloid peptides. The effects of LPE on the fibrillation of A*β* peptides were measured by monitoring Th-T fluorescence emission. A*β*_1–40_ (100 μM) incubated in 0.2 M phosphate buffer, pH 8.0, in the presence of increasing LPE concentrations for 24 h at 37 °C. Data are expressed as percentage vs. control samples (in absence of LPE) and interpolated by a nonlinear fitting in a hyperbolic function (r^2^ = 0.995).

Therefore, we evaluated the ability of LPE to affect molecular interactions of A*β*_1–40_ amyloid peptides during the aggregation process by an in vitro assay, as detailed in the Methods section. In this assay, the formation of fibrils was performed in the absence or in the presence of increasing LPE concentrations for 24 h. The results (Figure 2) demonstrated that the presence of LPE inhibits the A*β*_1–40_ aggregation process in a dose-dependent manner. The data interpolated in a hyperbolic behavior allowed the calculation of an IC_50_ value corresponding to 176.01 ± 7.57 μM.

### 2.3. Effect of LPE on MAO-A and MAO B Activity

**Figure 3:**
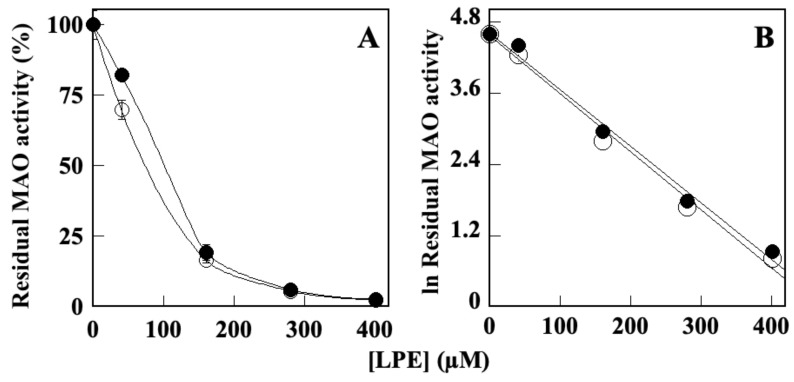
Effect of LPE on in vitro MAO-A and MAO-B activity. (**A**) The MAO-A (black circle) and MAO-B (open circle) enzyme activities were assayed in the presence or in the absence of the indicated amounts of LPE and reported as percentage of that measured in the absence of LPE. (**B**) Data were analyzed according to a first-order behavior and the IC_50_ values, were calculated from the slope of the linear regression (r^2^ = 0.991 for MAO-A and r^2^ = 0.992 for MAO-B).

Since overexpression of MAOs has been observed in AD and PD, leading to neuronal death and brain disfunctions, MAO inhibitors are currently approved drugs used to treat neurodegenerative diseases, although with significative side effects [18,68]. The ability of LPE to inhibit MAO-A and MAO-B activity was explored by in vitro enzyme assays [51,69], performed in the absence or presence of increasing LPE concentrations. The results (Figure 3) indicate that LPE can inhibit either MAO-A or MAO-B activity (Figure 3A), with IC_50_ values corresponding to 81.6 ± 1.2 µM (r^2^ = 0.991) and 78.3 ± 3.6 µM (r^2^ = 0.992), respectively, as calculated by the semilogarithmic plots (Figure 3B). It must be pointed out that, under the same experimental conditions, the two specific MAO-A and MAO-B inhibitors, clorgylin and selegiline, respectively, showed an IC_50_ of 150 ± 22 nM and 230 ± 48 nM [51].

### 2.4. Effect of LPE on SOD Activity

**Figure 4:**
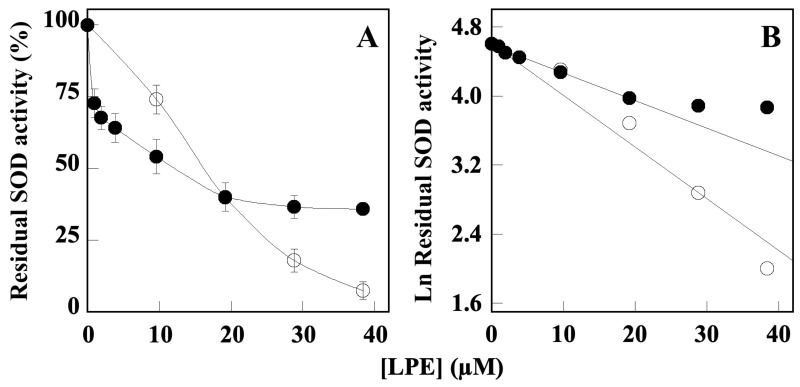
Effect of LPE on in vitro Cu/Zn SOD (SOD1) and Mn SOD (SOD2) enzyme activity. (**A**) The SOD1 (black circle) and SOD2 (open circle) enzyme activities were assayed in the presence or in the absence of the indicated amounts of LPE, ranging from 40 to 400 µM GAE. (**B**) Data were analyzed according to a first order behavior and the IC_50_ values were calculated from the slope of the linear regression (r^2^ = 0.9913 for SOD1 and r^2^ = 0.9925 for SOD2). Data are expressed as percentage vs. control samples (in absence of LPE) ± SD.

Because the production of ROS contributes either to the impairment of cholinergic and aminergic neurotransmission or formation of the Aβ-amyloid plaques and Tau fibrils, we also evaluated the effect of LPE on SOD enzymes that play a crucial role in the cell system of antioxidant defense. Figure 4 shows the ability of LPE to inhibit both SOD1 and SOD2 activity by in vitro enzyme assay that was performed in the absence or in the presence of increasing concentration of LPE. These results demonstrated the LPE-dependent enzyme inhibition, with a calculated IC_50_ value corresponding to 21.50 ± 3.20 µM for SOD1 and 10.02 ± 1.30 µM for SOD2. Although both enzymes show a similar sensibility to LPE inhibition, the inactivation profile showed by SOD2 could suggest a co-operative inhibition mechanism.

## 3. Discussion

Epidemiological studies have demonstrated that diets rich in polyphenols, such as the Mediterranean diet, can prevent and ameliorate symptoms of cognitive decline and neurodegenerative disorders, such as Alzheimer’s and Parkinson’s diseases that affect mostly the elderly population [46,70,71]. Moreover, the high polyphenol content apported by fruits and vegetables of the Mediterranean diet has also been correlated to a reduced inflammation status of the body and neuroprotection [72]. *Citrus lemon* is a fruit abundant in the Mediterranean diet and it is rich in vitamins and flavonoids that show many antioxidant and beneficial effects against inflammation and cancer [52,61,67]. In addition, *Citrus* peels represent a useful source for the recovery of biological active polyphenols that possess radical scavenging properties and contain neuroprotective components [73,74,75].

In this article, we describe novel biochemical properties of LPE obtained from the waste of the agro-food industry of *Citrus lemon* peels, suggesting its potential use in the prevention and amelioration of the symptoms of neurodegenerative disorders such as AD and PD. Although AD and PD are multifactorial neurodegenerative disorders, they share common molecular and biochemical features, including key enzymes involved in the neurotransmitter’s metabolism.

The main clinical features of AD are the decrease in ACh concentration following the death of cholinergic neurons caused by the formation of A*β* protein plaques. In the brain, ACh is degraded by AChE and by BuChE, although with less efficiency.

To date, the main therapeutic approach in the management of AD is based on the use of cholinesterase inhibitors, such as tacrine, donepezil, rivastigmine, and galantamine, that also inhibit the formation of A*β* plaques, which counteract the decrease in the concentration of ACh [76]. Currently, the drugs most used in therapy are donepezil and rivastigmine, which fail to reverse the causes but only alleviate the symptoms and, unfortunately, induce side effects at the hepatic and gastro-intestinal level [36]. In addition, because neurodegenerative disorders are multifactorial diseases, multitarget therapeutic approaches and combination therapy have been developed, aiming not only to restore the concentration of neurotransmitters, but also reducing or preventing protein aggregation, such as A*β* peptide, Tau fibrillary tangles, reduction in inflammatory cytokines and oxidative stress conditions.

For these reasons, research has aimed at the identification of natural inhibitory molecules present in the diet such as polyphenols, which possess neuroprotective activities [40,46,55,72,77] and are able to cross the blood–brain barrier [59]. Among the various dietary polyphenols, numerous studies conducted in vivo and in vitro have demonstrated that aqueous extracts of citrus peel, including lemon, are rich in phenolic compounds, in particular flavonoids, which not only inhibit AChE, but also BuChE activity [78,79] and MAO [52]. The previous characterization of LPE has demonstrated that it shows antioxidant properties [67], anti-inflammatory and chemo-preventive activity in human gastric and colon cancer cells and inhibits in vitro AChE activity [60,61]. Further, we also show that LPE inhibits the enzymatic activity of the other two key enzymes involved in the metabolism of cholinergic (ACh) and dopaminergic (biogenic amines) neurotransmitters that involve, respectively, BuChE and MAO-A/B activity. Although AChE is the major target enzyme in the studies aiming to develop drugs against AD, inhibition of BuChE has also been considered a key point in the treatment of AD. In fact, the concentration of BuChE increases during disease progression, leading to a decrease in ACh [80,81]. In this study, we demonstrated that LPE inhibits BuChE activity with an IC_50_ value (78 µM) higher than that determined of AChE (21 µM) [60]. Furthermore, the inhibitory effect on MAO is also of particular importance because an increase in MAOs activity has been found in the brain of AD patients, which causes a decrease in the concentration of monoamine neurotransmitters (dopamine, serotonin, and norepinephrine) [18,82]. AD and PD are multifactorial disorders also characterized by an increase in oxidative stress caused by inflammatory stimuli [24,25,26] and by an increase in MAO activity, leading to the generation of free radicals [83]. In fact, it is believed that the molecules capable of inhibiting the formation of H_2_O_2_ and NH_3_, derived from the degradation of the MAO amines, are the basis of their protective effect [84]. MAO inhibition may be related to phenolic composition because some derivatives of flavonoid inhibiting MAOs are structurally related to synthetic inhibitors [39].

The biological effects exerted by flavonoids include their antioxidant activity through the direct scavenging of ROS and metal chelating property, as well as the modulation of enzymes acting in the endogenous antioxidant system. SODs are among the main enzymes of antioxidant defense system that catalyzes the dismutation’s reaction of superoxide anion (O_2_^•−^), thus reducing further generation of free radicals. However, the reaction catalyzed by SODs leads to the production of H_2_O*_2_*, thus requiring the action of catalase for its removal [27]. Our results (Figure 4) indicated that LPE inhibit either SOD1 or SOD2 in a concentration-dependent manner and with a similar sensitivity to LPE (IC_50_ values of 21.50 ± 3.20 µM and 10.02 ± 1.30 µM, respectively), suggesting a co-operative inhibition mechanism for the inactivation profile of SOD2. Although the molecular mechanism of SOD1 and SOD2 inhibition by LPE needs further investigation, this result prompts us to suppose that this effect could reduce the production of H_2_O_2_.

Biochemical properties exerted by LPE may be attributed to its chemical composition that shows as major compounds naringenin and quercetin rutinoside (6.53 and 0.923 mg/g, respectively) (Supplementary Figure S1) [67]. These results agree with those obtained in previous studies on naringenin and quercetin rutinoside tested as single components, although we cannot compare the concentrations of polyphenols used in these studies derived by different in vitro and in vivo models [54,55,56,57,58]. Furthermore, we cannot exclude that less abundant compounds in LPE, such as naringin, *p*-Coumaric acid, and hesperitin [67], can also exert additive or synergic effect. Taken together, these results, summarized in Table 1, suggest that LPE behaves as a multitargeting agent against key factors involved in AD and PD, through the inhibition to varying extents of ChE, MAOs, SODs, and its ability to reduce A*β*-fibril aggregation.

Although the results obtained on the neuroprotective properties of LPE are highly encouraging, the data obtained cannot be directly translated in a cell context, as this study concerns the use of an in vitro system model. In fact, LPE has been tested on purified enzymes and on amyloid beta fibrillation process in the absence of specific receptors and cellular components. Using this method, LPE components directly interact with binding and/or regulatory sites of a specific enzyme domain(s) or they can directly affect the fibril formation via intermolecular interactions.

However, this methodology limitation may also be advantageous because it allows further studies on the interactions and molecular mechanisms that trigger either enzyme inhibition or fibril formation.

In fact, this in vitro model may allow the identification of the specific LPE component(s) interacting with the amyloid enzyme/peptide domains, as well as the intermolecular interacting domains, using advanced biochemical methodology. Moreover, synergic effects of more than one component cannot be excluded.

## 4. Materials and Methods

### 4.1. Materials

Butyrylcholinesterase from equine serum (BuChE), butyrylthiocholine, 5′,5′-dithiobis-2-nitrobenzoic acid (DTNB), human monoamine oxidase A and B, Cu/Zn SOD from bovine erythrocytes, kynuramine, donepezil, clorgylin, selegiline, and thioflavine T were purchased from Sigma-Aldrich (Milano, Italy). Human A*β*_1–40_ amyloid peptide (cat. Ab120479) was obtained by Abcam (Cambridge, UK). Purified recombinant Mn SOD was obtained in Streptococcus mutans as reported [85].

### 4.2. Methods

#### 4.2.1. Preparation of Lemon Peel Polyphenol Extract (LPE) and Determination of Total Polyphenol Content

*Citrus limon* peels, derived from vegetable waste for liquor production, were kindly supplied by Villa Massa (Piano di Sorrento, NA, Italy). LPE was obtained as previously described [60,67]. Briefly, the peels were frozen at −20 °C and then lyophilized into a freeze-dry system (Labconco, 12 Liter Console Freeze Dry System, Kansas City, MO, USA). The dry solid material was sequentially ground to obtain a homogenous fine powder (particle size of about 1 mm). Polyphenols were recovered by maceration as follows: 1 g of powdered waste was suspended in 25 mL of 80% EtOH and left under stirring at room temperature for 2 h. Partial purification was performed by discarding the resting solid material by centrifugation at 10,000 rpm for 40 min (Centrifuge Avanti™ J-25, rotor JA14 Beckman Coulter™). The liquid phase was filtered on paper filter (Whatman^®^ cellulose Grade 1) and successively reduced to 10–15 mL in a rotary evaporator (Buchi Rotavapor R-210) at 40 °C under vacuum. The raw extract was concentrated under a liquid nitrogen stream, and then the pellet was resuspended in the same volume of PBS. The concentration of total phenolic content was determined according to an adapted Folin–Ciocalteu colorimetric method [86], and the results were expressed as molar Gallic Acid Equivalent (GAE) per g of dry sample. Thereafter, LPE was aliquoted and stored at −20 °C until it was used. The chemical composition of LPE previously reported [67] was confirmed by reverse-phase (RP)–high-performance liquid chromatography (HPLC) (Supplementary Figure S1).

#### 4.2.2. Butyrylcholinesterase In Vitro Enzyme Assay

Butyrylcholinesterase (BuChE) activity was assayed by the Ellman method [87] using butyrylthiocoline as substrate. The reduction of dithio-bis-nitrobenzoate by the thiocholine, produced by the enzymatic hydrolysis of the thiolated substrate, was followed colorimetrically (412 nm) at room temperature (22–25 °C) using a Cary 100 spectrophotometer (Agilent, Milan, Italy). The reaction mixture (500 µL) containing 330 µM 5,5′-dithio-bis-2-nitrobenzoic acid (DTNB) and 500 µM butyrylthiocoline as substrate was prepared in 0.1 M sodium phosphate buffer, pH 7.4, in the absence or in the presence of different amounts of LPE. The reaction was started by the addition of 100 mU/mL BuChE, and the initial rate of the reaction was derived from the linear portion of the kinetics. The concentration of LPE required to reduce the enzymatic activity to 50% (IC_50_) was derived from semi-logarithmic plots in which the logarithm of the residual activity was plotted against the LPE concentration. Linear curve fits were obtained with the least-squares method, and the significance of the correlation was estimated from the squared correlation coefficient r^2^.

#### 4.2.3. A*β*_1–40_ Self-Aggregation Inhibition Assay

A*β*_1–40_ self-aggregation was performed as previously reported [11]; briefly, 96 µM peptide in 12 µL of 200 mM sodium phosphate buffer (pH 8.0) containing 0.5% (*v*/*v*) DMSO at 37 °C for 24 h in the absence or in the presence of increasing concentrations of LPE. To quantify amyloid fibril formation, 0.5 mL of 1.6 µM thioflavine T in 50 mM Glycine-NaOH buffer (pH 8.5) was added. Therefore, a time scan of fluorescence intensity (300 s) was carried out using an excitation and emission wavelength of 446 and 490 nm, respectively (slits were set to 10 nm for both the excitation and the emission beam), using a Cary-Eclipse spectrofluorimeter (Agilent, Milan, Italy); the fluorescence values at plateau were averaged over an at least 2 min scan. The residual self-aggregation inhibition was calculated from the decrease in the fluorescence signal after the subtraction of the background fluorescence of a thioflavin T solution obtained in the same way. The concentration leading to 50% residual self-aggregation (SA50) was derived from a nonlinear fitting of the data in a hyperbolic function in which the logarithm of the residual self-aggregation was plotted against the LPE concentration.

#### 4.2.4. Monoamine Oxidase Assay

Monoamine oxidase activity was assayed by the fluorimetric method previously reported [69]. This method was based on the oxidation of kynuramine by monoamine oxidase that led to the production of 8-hydroxychinoline, which becomes fluorescent in alkaline conditions. The 250 μL reaction mixture was prepared in a 50 mM potassium phosphate buffer, pH 7.1, and contained 40 μM kynuramine in the absence or presence of different concentrations of LPE. The reaction was started by adding monoamine oxidase A or B (3.75 μg) and allowed to proceed for 20 min. The enzymatic oxidation of the substrate was stopped by adding 150 μL of 2 M NaOH and, after 10 min incubation at room temperature, 240 μL of water. The resulting mixture was centrifuged for 10 min at 15,000 rpm and the fluorescence was measured on 500 μL of the supernatant using a Cary Eclipse Spectrofluorimeter (Agilent, Milan, Italy). The fluorescence signal was recorded at room temperature (20–25 °C) using an excitation and emission wavelength of 315 and 380 nm, respectively; slits were set to 10 nm for both the excitation and the emission beam. The residual activity was referred to that measured in the absence of LPE, and the data were collected in at least three different experiments. The concentration leading to 50% residual activity (IC_50_) was derived from a semilogarithmic plot in which the logarithm of the residual activity was plotted against inhibitor concentration.

#### 4.2.5. SOD Inhibition Assay

SOD activity was measured at 25 °C in 100 mM potassium phosphate buffer, pH 7.8, containing 0.1 mM Na-EDTA by the inhibition of cytochrome c reduction caused by superoxide anions generated with the xanthine/xanthine oxidase method, as previously reported [85,88].

#### 4.2.6. Statistical Analysis

The significance of the correlation in linear and hyperbolic curve fits was estimated by the squared correlation coefficient r^2^. The data were expressed as mean ± SD of at least three independent experiments performed in triplicate. Analyses were carried out using version 5.0 of the KaleidaGraph program (Synergy, Adalta, Italy).

## 5. Conclusions

The data reported in this article highlight the ability of LPE to inhibit key enzymes and the A*β*_1–40_ fibril aggregation. LPE behaves as a multitargeting agent for its ability to interact with multiple factors underlying AD and PD diseases. LPE represents a useful nonpharmacological and a diet-based approach to prevent or delay the insurge and counteract these neurodegenerative disorders. These biochemical properties make LPE a promising candidate as integrative supplementation of pharmacological treatment in the prevention and management of AD and PD.

These data suggest further development of LPE characterization for its potential use in neuroprotection, even considering a validation by additional in vitro as well as in vivo models.

Further application of LPE can be devoted in the development of functional foods involving also the recovery of biological active polyphenols from lemon peel agroindustry wastes.

## Figures and Tables

**Table 1:** IC_50_ values of LPE inhibition on enzymes and A*β*_1–40_ fibril formation.

Enzyme	LPE IC_50_ (µM) ± SD *
BuChE	72.9 ± 1.5
MAO-A	81.6 ± 1.2
MAO-B	78.3 ± 3.6
SOD1	21.5 ± 3.2
SOD2	10.0 ± 1.3
**Fibrillation process**	
A*β*_1–40_ fibril formation	176.0 ± 7.6

* Values are expressed as mean ± SD calculated on at least three different determinations.

## Data Availability

Data are contained within the article.

## Supplementary Materials

The following supporting information can be downloaded at: https://www.mdpi.com/article/10.3390/molecules28176332/s1: Figure S1: HPLC profile of LPE.

## References

[1] Wyss-Coray T. (2016). Ageing, neurodegeneration and brain rejuvenation. Nature.

[2] Mayne K., White J.A., McMurran C.E., Rivera F., de la Fuente A.G. (2020). Aging and Neurodegenerative Disease: Is the Adaptive Immune System a Friend or Foe?. Front. Aging Neurosci..

[3] Sheikh S., Haque E., Mir S.S. (2013). Neurodegenerative Diseases: Multifactorial Conformational Diseases and Their Therapeutic Interventions. J. Neurodegen. Dis..

[4] Chen X.Q., Mobley W.C. (2019). Alzheimer disease pathogenesis: Insights from molecular and cellular biology studies of oligomeric abeta and tau species. Front. Neurosci..

[5] Francis P.T., Palmer A.M., Snape M., Wilcock G.K. (1999). The cholinergic hypothesis of Alzheimer’s disease: A review of progress. J. Neurol. Neurosurg. Psychiatry.

[6] Ferreira-Vieira T.H., Guimaraes I.M., Silva F.R., Ribeiro F.M. (2016). Alzheimer’s disease: Targeting the Cholinergic System. Curr. Neuropharmacol..

[7] Arcone R., Chinali A., Pozzi N., Parafati M., Maset F., Pietropaolo C., De Filippis V. (2009). Conformational and Biochemical Characterization of a Biologically Active Rat Recombinant Protease Nexin-1 Expressed in *E. coli*. Biochim. Biophys. Acta.

[8] Alcaro S., Arcone R., Costa G., De Vita D., Iannone M., Ortuso F., Procopio A., Pasceri R., Rotiroti D., Scipione L. (2010). Simple choline esters as potential anti-Alzheimer agents. Curr. Pharm. Des..

[9] Birks J.S., Evans J.G. (2015). Rivastigmine for Alzheimer’s disease. Cochrane Database System. Rev..

[10] Costanzo P., Cariati L., Desiderio D., Sgammato R., Lamberti A., Arcone R., Salerno R., Nardi M., Masullo M., Oliverio M. (2016). Design, Synthesis, and Evaluation of Donepezil-Like Compounds as AChE and BACE-1 Inhibitors. ACS Med. Chem. Lett..

[11] Vitale R.M., Rispoli V., Desiderio D., Sgammato R., Thellung S., Canale C., Vassalli M., Carbone M., Ciavatta M.L., Mollo E. (2018). In Silico Identification and Experimental Validation of Novel Anti-Alzheimer’s Multitargeted Ligands from a Marine Source Featuring a “2-Aminoimidazole plus Aromatic Group” Scaffold. ACS Chem. Neurosci..

[12] Zhou Y., Lu X., Yang H., Chen Y., Wang F., Li J., Tang Z., Cheng X., Yang Y., Xu L. (2019). Discovery of Selective Butyrylcholinesterase (BChE) Inhibitors through a Combination of Computational Studies and Biological Evaluations. Molecules.

[13] Eriksen J.L., Wszolek Z., Petrucelli L. (2005). Molecular pathogenesis of Parkinson disease. Arch. Neurol..

[14] Shih J.C., Thompson R.F. (1999). Monoamine oxidase in neuropsychiatry and behavior. Am. J. Hum. Genet..

[15] Manzoor S., Hoda N. (2020). A comprehensive review of monoamine oxidase inhibitors as Anti-Alzheimer’s disease agents: A review. Eur. J. Med. Chem..

[16] Naoi M., Maruyama W., Inaba-Hasegawa K. (2012). Type A and B monoamine oxidase in age-related neurodegenerative disorders: Their distinct roles in neuronal death and survival. Curr. Top. Med. Chem..

[17] Shih J.C., Chen K., Ridd M.J. (1999). Monoamine oxidase: From genes to behavior. Ann. Rev. Neurosci..

[18] Cho H.U., Kim S., Sim J., Yang S., An H., Nam M.H., Jang D.P., Lee C.J. (2021). Redefining differential roles of MAO-A in dopamine degradation and MAO-B in tonic GABA synthesis. Exp. Mol. Med..

[19] Schedin-Weiss S., Inoue M., Hromadkova L., Teranishi Y., Yamamoto N.G., Wiehager B., Bogdanovic N., Winblad B., Sandebring-Matton A., Frykman S. (2017). Monoamine oxidase B is elevated in Alzheimer disease neurons, is associated with γ-secretase and regulates neuronal amyloid β-peptide levels. Alzheimers Res. Ther..

[20] Chaurasiya N.D., Leon F., Muhammad I., Tekwani B.L. (2022). Natural Products Inhibitors of Monoamine Oxidases-Potential New Drug Leads for Neuroprotection, Neurological Disorders, and Neuroblastoma. Molecules.

[21] Bhawna, Kumar A., Bhatia M., Kapoor A., Kumar P., Kumar S. (2022). Monoamine oxidase inhibitors: A concise review with special emphasis on structure activity relationship studies. Eur. J. Med. Chem..

[22] Wyss-Coray T. (2006). Inflammation in Alzheimer disease: Driving force, bystander or beneficial response?. Nat. Med..

[23] Avila-Muñoz E., Arias C. (2014). When astrocytes become harmful: Functional and inflammatory responses that contribute to Alzheimer’s disease. Ageing Res. Rev..

[24] Sofroniew M.V. (2009). Molecular dissection of reactive astrogliosis and glial scar formation. Trends Neurosci..

[25] Uttara B., Singh A.V., Zamboni P., Mahajan R.T. (2009). Oxidative stress and neurodegenerative diseases: A review of upstream and downstream antioxidant therapeutic options. Curr. Neuropharmacol..

[26] Ganguly U., Kaur U., Chakrabarti S.S., Sharma P., Agrawal B.K., Saso L., Chakrabarti S. (2021). Oxidative Stress, Neuroinflammation, and NADPH Oxidase: Implications in the Pathogenesis and Treatment of Alzheimer’s Disease. Oxidative Med. Cell. Longev..

[27] Maier C.M., Chan P.H. (2002). Book Review: Role of Superoxide Dismutases in Oxidative Damage and Neurodegenerative Disorders. Neuroscientist.

[28] Zelko I.N., Mariani T.J., Folz R.J. (2002). Superoxide dismutase multigene family: A comparison of the CuZn-SOD (SOD1), Mn-SOD (SOD2), and EC-SOD (SOD3) gene structures, evolution, and expression. Free Radic. Biol. Med..

[29] Case A.J. (2017). On the Origin of Superoxide Dismutase: An Evolutionary Perspective of Superoxide-Mediated Redox Signaling. Antioxidants.

[30] Ighodaro O.M., Akinloye O.A. (2018). First line defence antioxidants-superoxide dismutase (SOD), catalase (CAT) and glutathione peroxidase (GPX): Their fundamental role in the entire antioxidant defence grid. Alex. J. Med..

[31] Fridovich I. (1995). Superoxide radical and superoxide dismutases. Annu. Rev. Biochem..

[32] Liou W., Chang L.Y., Geuze H.J., Strous G.J., Crapo J.D., Slot J.W. (1993). Distribution of CuZn superoxide dismutase in rat liver. Free Radic. Biol. Med..

[33] Weisiger R.A., Fridovich I. (1973). Superoxide dismutase: Organelle specificity. J. Biol. Chem..

[34] Holley A.K., Bakthavatchalu V., Velez-Roman J.M., St Clair D.K. (2011). Manganese superoxide dismutase: Guardian of the powerhouse. Int. J. Mol. Sci..

[35] Marklund S.L., Holme E., Hellner L. (1982). Superoxide dismutase in extracellular fluids. Clin. Chim. Acta.

[36] Tuzimski T., Petruczynik A. (2022). Determination of Anti-Alzheimer’s Disease Activity of Selected Plant Ingredients. Molecules.

[37] Costanzo P., Oliverio M., Maiuolo J., Bonacci S., De Luca G., Masullo M., Arcone R., Procopio A. (2021). Novel Hydroxytyrosol-Donepezil Hybrids as Potential Antioxidant and Neuroprotective Agents. Front. Chem..

[38] El-Hussieny M., Abd-El-Maksoud M.A., Soliman F.M., Fouad M.A., El-Ashrey M.K. (2023). Dual-target ligand discovery for Alzheimer’s disease: Triphenylphosphoranylidene derivatives as inhibitors of acetylcholinesterase and β-amyloid aggregation. J. Enzym. Inhib. Med. Chem..

[39] Benamar H., Rached W., Derdour A., Marouf A. (2010). Screening of Algerian medicinal plants for acetylcholinesterase inhibitory activity. J. Biol. Sci..

[40] Nishal S., Phaugat P., Bazaad J., Dhaka R., Khatkar S., Khatkar A., Khayatkashani M., Alizadeh P., Haghighi S.M., Mehri M. (2023). A Concise Review of Common Plant-derived Compounds as a Potential Therapy for Alzheimer’s Disease and Parkinson’s Disease: Insight into Structure-Activity-Relationship. CNS Neurol. Disord. Drug Targets.

[41] Chimenti F., Secci D., Bolasco A., Chimenti P., Bizzarri B., Granese A., Carradori S., Yanez M., Orallo F., Ortuso F. (2009). Synthesis, molecular modeling, and selective inhibitory activity against human monoamine oxidases of 3-carboxamido-7-substituted coumarins. J. Med. Chem..

[42] Viña D., Serra S., Lamela M., Delogu G. (2012). Herbal natural products as a source of monoamine oxidase inhibitors: A review. Curr. Top. Med. Chem..

[43] Kong D., Yan Y., He X.Y., Yang H., Liang B., Wang J., He Y., Ding Y., Yu H. (2019). Effects of Resveratrol on the Mechanisms of Antioxidants and Estrogen in Alzheimer’s Disease. BioMed Res. Int..

[44] Li J., He Y., Fu J., Wang Y., Fan X., Zhong T., Zhou H. (2023). Dietary supplementation of *Acanthopanax senticosus* extract alleviates motor deficits in MPTP-induced Parkinson’s disease mice and its underlying mechanism. Front. Nutr..

[45] Balakrishnan R., Azam S., Cho D.-Y., Su-Kim I., Choi D.K. (2021). Natural Phytochemicals as Novel Therapeutic Strategies to Prevent and Treat Parkinson’s Disease: Current Knowledge and Future Perspectives. Oxidative Med. Cell. Longev..

[46] Carregosa D., Mota S., Ferreira S., Alves-Dias B., Loncarevic-Vasiljkovic N., Crespo C.L., Menezes R., Teodoro R., Santos C.N.D. (2021). Overview of Beneficial Effects of (Poly)phenol Metabolites in the Context of Neurodegenerative Diseases on Model Organisms. Nutrients.

[47] Caruso F., Incerpi S., Pedersen J., Belli S., Kaur S., Rossi M. (2022). Aromatic Polyphenol π-π Interactions with Superoxide Radicals Contribute to Radical Scavenging and Can Make Polyphenols Mimic Superoxide Dismutase Activity. Curr. Issues Mol. Biol..

[48] Zandi P.P., Anthony J.C., Khachaturian A.S., Stone S.V., Gustafson D., Tschanz J.T., Norton M.C., Welsh-Bohmer K.A., Breitner J.C. (2004). Cache County Study Group. Reduced risk of Alzheimer disease in users of antioxidant vitamin supplements: The Cache County Study. Arch. Neurol..

[49] Gardener H., Caunca M.R. (2018). Mediterranean Diet in Preventing Neurodegenerative Diseases. Curr. Nutr. Rep..

[50] Cannataro R., Fazio A., La Torre C., Caroleo M.C., Cione E. (2021). Polyphenols in the Mediterranean Diet: From Dietary Sources to microRNA Modulation. Antioxidants.

[51] Nasso R., Pagliara V., D’Angelo S., Rullo R., Masullo M., Arcone R. (2021). Annurca Apple Polyphenol Extract Affects Acetyl-Cholinesterase and Mono-Amine Oxidase In vitro Enzyme Activity. Pharmaceuticals.

[52] Ademosun A.O., Oboh G. (2014). Anticholinesterase and antioxidative properties of water-extractable phytochemicals from some citrus peels. J. Basic. Clin. Physiol. Pharmacol..

[53] Ademosun A.O. (2022). Citrus peels odyssey: From the waste bin to the lab bench to the dining table. Appl. Food Res..

[54] Seki T., Kamiya T., Furukawa K., Azumi M., Ishizuka S., Takayama S., Nagase S., Arai H., Yamakuni T., Yaegashi N. (2013). Nobiletin-rich Citrus reticulata peels, a kampo medicine for Alzheimer’s disease: A case series. Geriat. Gerontol. Int..

[55] Braidy N., Behzad S., Habtemariam S., Ahmed T., Daglia M., Nabavi S.M., Sobarzo-Sanchez E., Nabavi S.F. (2017). Neuroprotective Effects of Citrus Fruit-Derived Flavonoids, Nobiletin and Tangeretin in Alzheimer’s and Parkinson’s Disease. CNS Neurol. Disord. Drug Targets.

[56] Nakajima A., Aoyama Y., Nguyen T.T., Shin E.J., Kim H.C., Yamada S., Nakai T., Nagai T., Yokosuka A., Mimaki Y. (2013). Nobiletin, a citrus flavonoid, ameliorates cognitive impairment, oxidative burden, and hyperphosphorylation of tau in senescence-accelerated mouse. Behav. Brain Res..

[57] Wang D.M., Yang Y.J., Zhang L., Zhang X., Guan F.F., Zhang L.F. (2013). Naringin enhances CaMKII activity and improves long-term memory in a mouse model of Alzheimer’s disease. Int. J. Mol. Sci..

[58] Gopinath K., Sudhandiran G. (2012). Naringin modulates oxidative stress and inflammation in 3-nitropropionic acid-induced neurodegeneration through the activation of nuclear factor- erythroid 2-related factor-2 signalling pathway. Neurosci..

[59] Hwang S.L., Shih P.H., Yen G.C. (2012). Neuroprotective effects of citrus flavonoids. J. Agric. Food Chem..

[60] Pagliara V., Nasso R., Di Donato P., Finore I., Poli A., Masullo M., Arcone R. (2019). Lemon Peel Polyphenol Extract Reduces Interleukin-6-Induced Cell Migration, Invasiveness, and Matrix Metalloproteinase-9/2 Expression in Human Gastric Adenocarcinoma MKN-28 and AGS Cell Lines. Biomolecules.

[61] Pagliara V., De Rosa M., Di Donato P., Nasso R., D’Errico A., Cammarota F., Poli A., Masullo M., Arcone R. (2021). Inhibition of Interleukin-6-Induced Matrix Metalloproteinase-2 Expression and Invasive Ability of Lemon Peel Polyphenol Extract in Human Primary Colon Cancer Cells. Molecules.

[62] Pontifex M.G., Malik M.M.A.H., Connell E., Müller M., Vauzour D. (2021). Citrus Polyphenols in Brain Health and Disease: Current Perspectives. Front. Neurosci..

[63] Russo C., Maugeri A., Lombardo G.E., Musumeci L., Barreca D., Rapisarda A., Cirmi S., Navarra M. (2021). The Second Life of Citrus Fruit Waste: A Valuable Source of Bioactive Compounds. Molecules.

[64] Inaba-Hasegawa K., Shamoto-Nagai M., Maruyama W., Naoi M. (2017). Type B and A Monoamine Oxidase and Their Inhibitors Regulate the Gene Expression of Bcl-2 and Neurotrophic Factors in Human Glioblastoma U118MG Cells: Different Signal Pathways for Neuroprotection by Selegiline and Rasagiline. J. Neural. Transm..

[65] Alborghetti M., Nicoletti F. (2019). Different Generations of Type-B Monoamine Oxidase Inhibitors in Parkinson’s Disease: From Bench to Bedside. Curr. Neuropharmacol..

[66] Kumar B., Dwivedi A.R., Sarkar B., Gupta S.K., Krishnamurthy S., Mantha A.K., Parkash J., Kumar V. (2019). 4,6-Diphenylpyrimidine Derivatives as Dual Inhibitors of Monoamine Oxidase and Acetylcholinesterase for the Treatment of Alzheimer’s Disease. ACS Chem. Neurosci..

[67] Di Donato P., Taurisano V., Tommonaro G., Pasquale V., Jiménez J.M.S., de Pascual S., Poli T.A., Nicolaus B. (2017). Biological Properties of Polyphenols Extracts from Agro Industry’s Wastes. Waste Biomass Valor..

[68] Finberg J.P., Rabey J.M. (2016). Inhibitors of MAO-A and MAO-B in Psychiatry and Neurology. Front. Pharmacol..

[69] Saidemberg D.M., Ferreira M.A., Takahashi T.N., Gomes P.C., Cesar-Tognoli L.M., da Silva-Filho L.C., Tormena C.F., da Silva G.V., Palma M.S. (2009). Monoamine oxidase inhibitory activities of indolylalkaloid toxins from the venom of the colonial spider Parawixia bistriata: Functional characterization of PwTX-I. Toxicon.

[70] Spencer J.P.E. (2008). Food for thought: The role of dietary flavonoids in enhancing human memory, learning and neuro-cognitive performance. Proc. Nutr. Soc..

[71] Psaltopoulou T., Sergentanis T.N., Panagiotakos D.B., Sergentanis I.N., Kosti R., Scarmeas N. (2013). Mediterranean diet, stroke, cognitive impairment, and depression: A meta-analysis. Ann. Neurol..

[72] Mayr H.L., Thomas C.J., Tierney A.C., Kucianski T., George E.S., Ruiz-Canela M., Hebert J.R., Shivappa N., Itsiopoulos C. (2018). Randomization to 6-month Mediterranean diet compared with a low-fat diet leads to improvement in Dietary Inflammatory Index scores in patients with coronary heart disease: The AUSMED Heart Trial. Nutr. Res..

[73] Cirmi S., Ferlazzo N., Lombardo G.E., Ventura-Spagnolo E., Gangemi S., Calapai G., Navarra M. (2016). Neurodegenerative Diseases: Might *Citrus* Flavonoids Play a Protective Role?. Molecules.

[74] Furukawa Y., Okuyama S., Amakura Y., Sawamoto A., Nakajima M., Yoshimura M., Igase M., Fukuda N., Tamai T., Yoshida T. (2021). Isolation and Characterization of Neuroprotective Components from Citrus Peel and Their Application as Functional Food. Chem. Pharm. Bull..

[75] Matsuzaki K., Nakajima A., Guo Y., Ohizumi Y. (2022). A Narrative Review of the Effects of Citrus Peels and Extracts on Human Brain Health and Metabolism. Nutrients.

[76] Passos C.S., Simões-Pires C.A., Nurisso A., Soldi T.C., Kato L., de Oliveira C.M., de Faria E.O., Marcourt L., Gottfried C., Carrupt P.A. (2013). Indole alkaloids of Psychotria as multifunctional cholinesterases and monoamine oxidases inhibitors. Phytochemistry.

[77] Pagliara V., Parafati M., Adornetto A., White M.C., Masullo M., Grimaldi M., Arcone R. (2018). Dibutyryl cAMP- or Interleukin-6-induced astrocytic differentiation enhances mannose binding lectin (MBL)-associated serine protease (MASP)-1/3 expression in C6 glioma cells. Arch. Biochem. Bioph..

[78] Shahwar D., Rehman S.U., Raza M.A. (2010). Acetylcholinesterase inhibition potential and antioxidant activities of ferulic acid isolated from Impatiens bicolor Linn. J. Med. Plants Res..

[79] Szwajgier D., Borowiec K. (2012). Phenolic acids from malt are efficient acetylcholinesterase and butyrylcholinesterase inhibitors. J. Inst. Brew..

[80] Lane R.M., Potkin S.G., Enz A. (2006). Targeting acetylcholinesterase and butyrylcholinesterase in dementia. Int. J. Neuropsychopharmacol..

[81] Fernández-Bachiller M.I., Pérez C., Monjas L., Rademann J., Rodríguez-Franco M.I. (2012). New tacrine-4-oxo-4H-chromene hybrids as multifunctional agents for the treatment of Alzheimer’s disease, with cholinergic, antioxidant, and β-amyloid-reducing properties. J. Med. Chem..

[82] Adolfsson R., Gottfries C.G., Roos B.E., Winblad B. (1979). Changes in the brain catecholamines in patients with dementia of Alzheimer type. Br. J. Psychiatr..

[83] Matveychuk D., MacKenzie E.M., Kumpula D., Song M.S., Holt A., Kar S., Todd K.G., Wood P.L., Baker G.B. (2022). Overview of the Neuroprotective Effects of the MAO-Inhibiting Antidepressant Phenelzine. Cell Mol. Neurobiol..

[84] Ghasemi K., Ghasemi Y., Ebrahimzadeh M.A. (2009). Antioxidant activity, phenol and flavonoid contents of 13 citrus species peels and tissues. Pak. J. Pharm. Sci..

[85] De Vendittis A., Amato M., Mickniewicz A., Parlato G., De Angelis A., Castellano I., Rullo R., Riccitiello F., Rengo S., Masullo M. (2010). Regulation of the properties of superoxide dismutase from the dental pathogenic microorganism Streptococcus mutans by iron- and manganese-bound co-factor. Mol. BioSyst..

[86] Singleton V.L., Rossi J.A. (1965). Colorimetry of total phenolics with phosphomolybdic phosphotungstic acid reagents. Am. J. Enol. Viticult..

[87] Ellman G.L., Courtney K.D., Andres V., Featherstone R.M. (1961). A new and rapid colorimetric determination of acetylcholinesterase activity. Biochem. Pharmacol..

[88] Cerchia C., Roscetto E., Nasso R., Catania M.R., De Vendittis E., Lavecchia A., Masullo M., Rullo R. (2022). In Silico Identification of Novel Inhibitors Targeting the Homodimeric Interface of Superoxide Dismutase from the Dental Pathogen Streptococcus mutans. Antioxidants.

