# Supplementary material for: Inhibition of Enzymes Involved in Neurodegenerative Disorders and Aβ1–40 Aggregation by Citrus limon Peel Polyphenol Extract
Source: Molecules. 2023 Aug 30;28(17):6332. doi: 10.3390/molecules28176332 (PMC10489013; doi:10.3390/molecules28176332)
Supplement: Supplementary file 1 [file molecules-28-06332-s001.zip › molecules-2560958-supplementary.pdf]

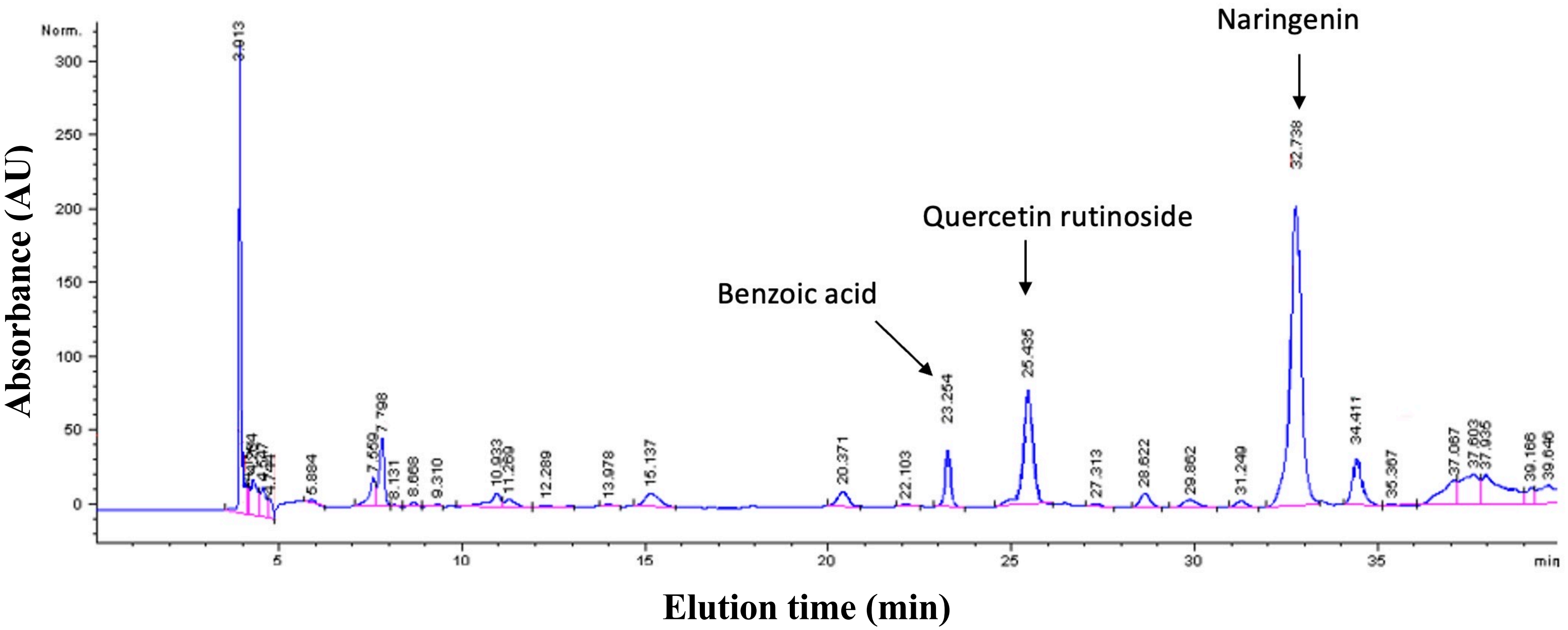

Supplementary Figure S1. RP-HPLC profile of LPE. Reverse phase (RP)-high performance liquid chromatogra- phy (HPLC) was carried out by means of a system equipped with an analytic Kromasyl C-18 column (250 × 4.6 mm, 5 µm particle sizes, Phenomenex) and a diode array detector (DAD, UV-2000), set at λ ranging from 280 to 350 nm. The column was eluted atroom temperature by a flow rate of 1.0 ml/min with a gradient of Solvent A (acetonitrile) and Solvent B (0.5% acetic acid) set as follows: 90/10 (B/A) from 0 to 10 min, 70/30 (B/A) from 10 to 40 min and 90/10 (B/A) from 40 to 50 min.

The main o-diphenols were identified on the basis of the retention time of authentic standard phenolic compounds naringenin, quercitin rutinoside and benzoic acid.
